# Supplementary material for: Single-nucleus multi-omics identifies shared and distinct pathways in Pick’s and Alzheimer’s disease
Source: Sci Adv. 2025 Nov 12;11(46):eads7973. doi: 10.1126/sciadv.ads7973 (PMC12609060; doi:10.1126/sciadv.ads7973)
Supplement: Supplementary file 1 — Supplementary Text Figs. S1 to S10 Legends for tables S1 to S5 Legend for data S1 [file sciadv.ads7973_sm.pdf]

Science Advances
AAAS

Supplementary Materials for

Single-nucleus multi-omics identifies shared and distinct pathways in Pick's and Alzheimer's disease

Zechuan Shi *et al.*

Corresponding author: Vivek Swarup, vswarup@uci.edu

Sci. Adv. **11**, eads7973 (2025)
DOI: 10.1126/sciadv.ads7973

The PDF file includes:

Supplementary Text
Figs. S1 to S10
Legends for tables S1 to S5
Legend for data S1

Other Supplementary Material for this manuscript includes the following:

Tables S1 to S5
Data S1

Supplementary Text

scROAD Interactive Database

We have developed scROAD (Single-cell Regulatory Occupancy Archive in Dementia), an interactive online resource designed to explore single-cell cCREs (candidate cis-regulatory elements) transcription factor occupancy data. This database provides comprehensive information derived from snATAC-seq analysis of human postmortem prefrontal cortex (PFC) tissue, with a specific focus on Alzheimer's Disease (AD) and Pick's Disease (PiD). scROAD enables researchers to perform exploratory searches for genes, transcription factors (TFs), and SNPs, as well as to visualize transcription factor regulatory networks. Additionally, users can download the data for their own research purposes.

Accessible at http://swaruplab.bio.uci.edu/scROAD, this resource is freely available for noncommercial research purposes. By providing high-resolution regulatory data, scROAD supports the broader scientific community in advancing our understanding of transcriptional regulation in dementia and fosters the generation of novel hypotheses and discoveries.

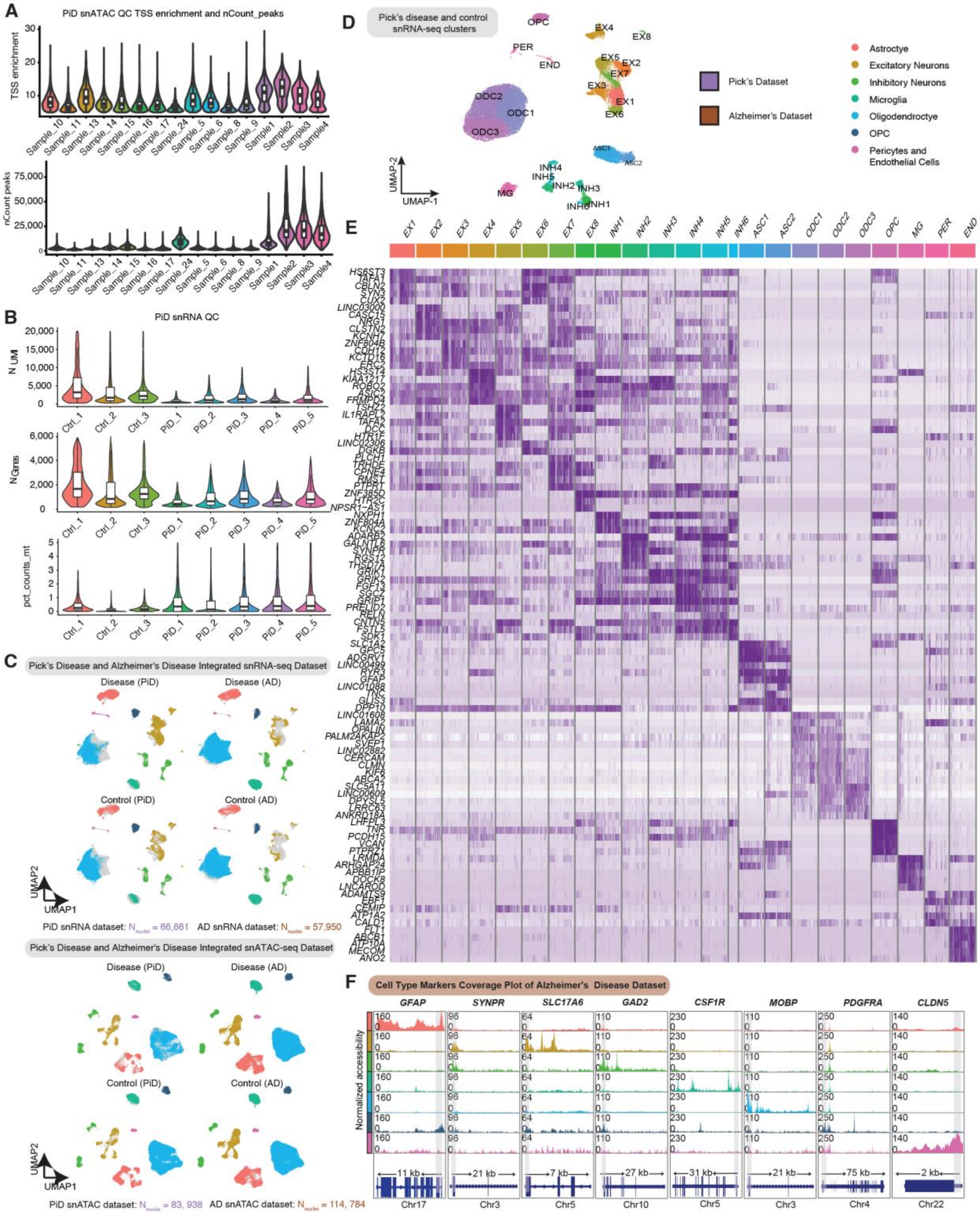

Fig. S1. Quality control and cell type annotations of the PiD and AD snMulti-omic datasets. (**A**) Violin plot showing the number of peak counts in the samples from the PiD PFC snATAC-seq dataset. (**B**) Violin plot showing the number of UMI, genes and mitochondrial percentage in

the samples from the PiD PFC snRNA-seq dataset. (**C**) Integrated Uniform Manifold Approximation and Projection (UMAP) visualizations by diagnosis for snRNA-seq and snATAC-seq data from PiD and AD. (**D**) Uniform Manifold Approximation and Projection (UMAP) visualizations for clusters of snRNA-seq data from PiD. (**E**) Heatmap of canonical cell-type markers for snRNA-seq data from PiD. (**F**) Coverage plots for canonical cell-type markers in AD dataset: *GFAP* (chr17:44905000-44916000) for astrocytes, *SYNPR* (chr3:63278010-63278510) for neurons, *SLC17A6* (chr11:22338004-22345067) for excitatory neurons, GAD2 (chr10:26214210-26241766) for inhibitory neurons, *CSF1R* (chr5:150056500-150087500) for microglia, *MOBP* (chr3:39467000-39488000) for oligodendrocytes, *PDGFRA* (chr4:54224871-54300000) for Pericytes and Endothelial cells in PiD dataset. The gray bar within each box highlights the promoter regions.

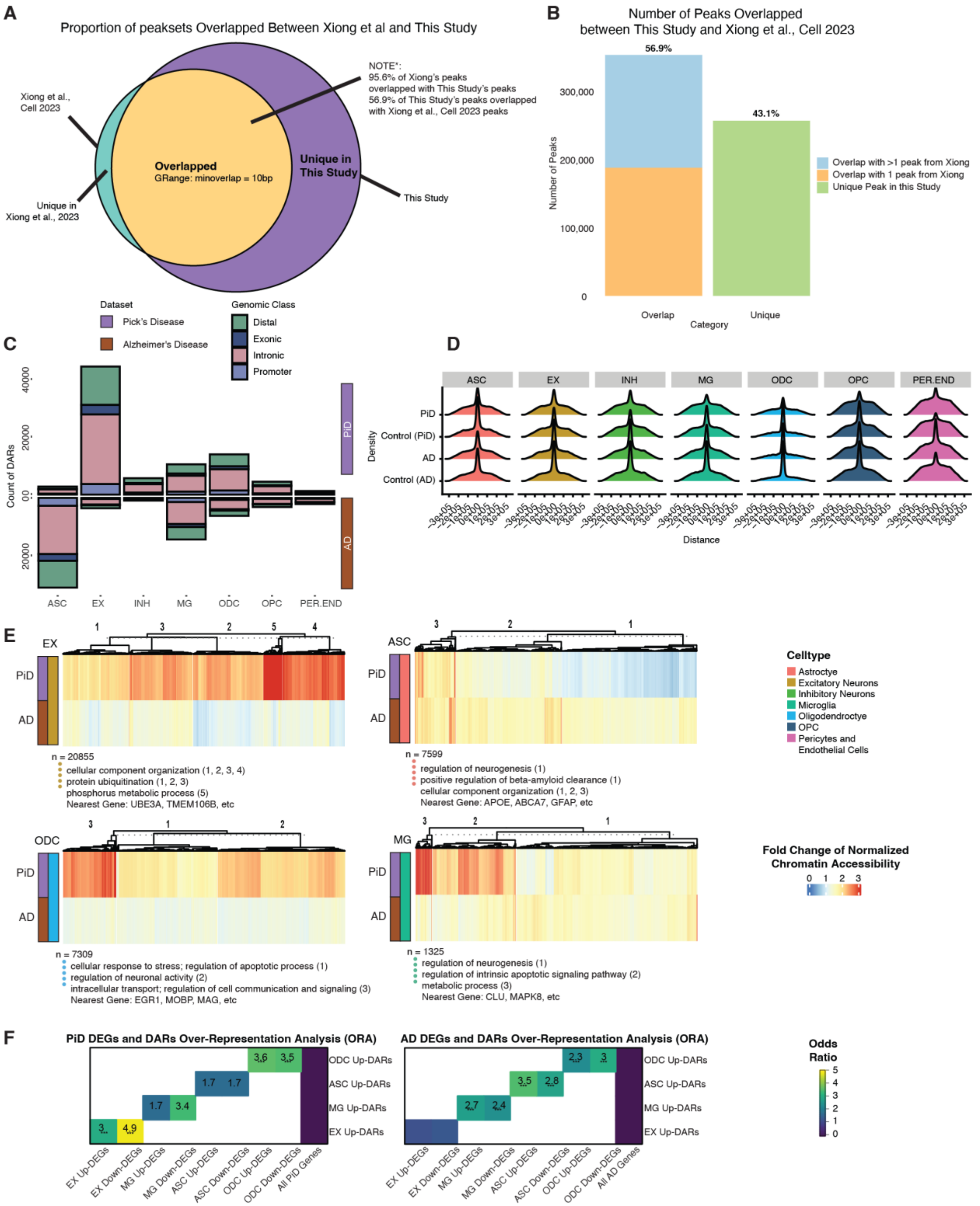
A
Proportion of peaksets Overlapped Between Xiong et al and This Study
Xiong et al., Cell 2023
NOTE*: 95.6% of Xiong's peaks overlapped with This Study's peaks 56.9% of This Study's peaks overlapped with Xiong et al., Cell 2023 peaks
Overlapped
GRange: minoverlap = 10bp
Unique in This Study
This Study
Unique in Xiong et al., 2023
B
Number of Peaks Overlapped between This Study and Xiong et al., Cell 2023
56.9%
43.1%
Number of Peaks
Overlap with >1 peak from Xiong
Overlap with 1 peak from Xiong
Unique Peak in this Study
Overlap
Unique
Category
C
Dataset
Pick's Disease
Alzheimer's Disease
Genomic Class
Distal
Exonic
Intronic
Promoter
Count of DARs
PiD
AD
ASC
EX
INH
MG
ODC
OPC
PER.END
D
PiD
Control (PiD)
AD
Control (AD)
Density
Distance
E
n = 20855
cellular component organization (1, 2, 3, 4)
protein ubiquitination (1, 2, 3)
phosphorus metabolic process (5)
Nearest Gene: UBE3A, TMEM106B, etc
n = 7599
regulation of neurogenesis (1)
positive regulation of beta-amyloid clearance (1)
cellular component organization (1, 2, 3)
Nearest Gene: APOE, ABCA7, GFAP, etc
Celltype
Astroctye
Excitatory Neurons
Inhibitory Neurons
Microglia
Oligodendroctye
OPC
Pericytes and Endothelial Cells
n = 7309
cellular response to stress; regulation of apoptotic process (1)
regulation of neuronal activity (2)
intracellular transport; regulation of cell communication and signaling (3)
Nearest Gene: EGR1, MOBP, MAG, etc
n = 1325
regulation of neurogenesis (1)
regulation of intrinsic apoptotic signaling pathway (2)
metabolic process (3)
Nearest Gene: CLU, MAPK8, etc
Fold Change of Normalized Chromatin Accessibility
F
PiD DEGs and DARs Over-Representation Analysis (ORA)
AD DEGs and DARs Over-Representation Analysis (ORA)
ODC Up-DARs
ASC Up-DARs
MG Up-DARs
EX Up-DARs
EX Up-DEGs
EX Down-DEGs
MG Up-DEGs
MG Down-DEGs
ASC Up-DEGs
ASC Down-DEGs
ODC Up-DEGs
ODC Down-DEGs
All PiD Genes
All AD Genes
Odds Ratio

Fig. S2. Comprehensive analysis of peak overlaps, cis-regulatory construction and changes in PiD and AD.

(**A**) Proportion of peaksets overlapped between Xiong et al. (*27*) and this study. (**B**) Number of peaks overlapped between this study and Xiong et al. (**C**) Genomic type classification of differential open accessible regions grouped by cell types (P-value < 0.05) between PiD and AD with their respective controls. (**D**) Ridgeline plot showing the distance of imputed enhancers from the promoters. (**E**) Heatmaps of fold changes (Disease vs. Control) on normalized chromatin accessibility of differential accessible intronic regions in excitatory neurons, astrocytes, microglia and oligodendrocytes (FDR adjusted P-value < 0.05 and abs(log2FC) > 0.5), gene ontology acquired from GREAT and examples of promoters and distal regions' cis-regulatory linked gene as in the panel of Fig. 2E. (**F**) Over-representation analysis (ORA) of DEGs (snRNA-seq) and DARs (snATAC-seq) from PiD and AD.

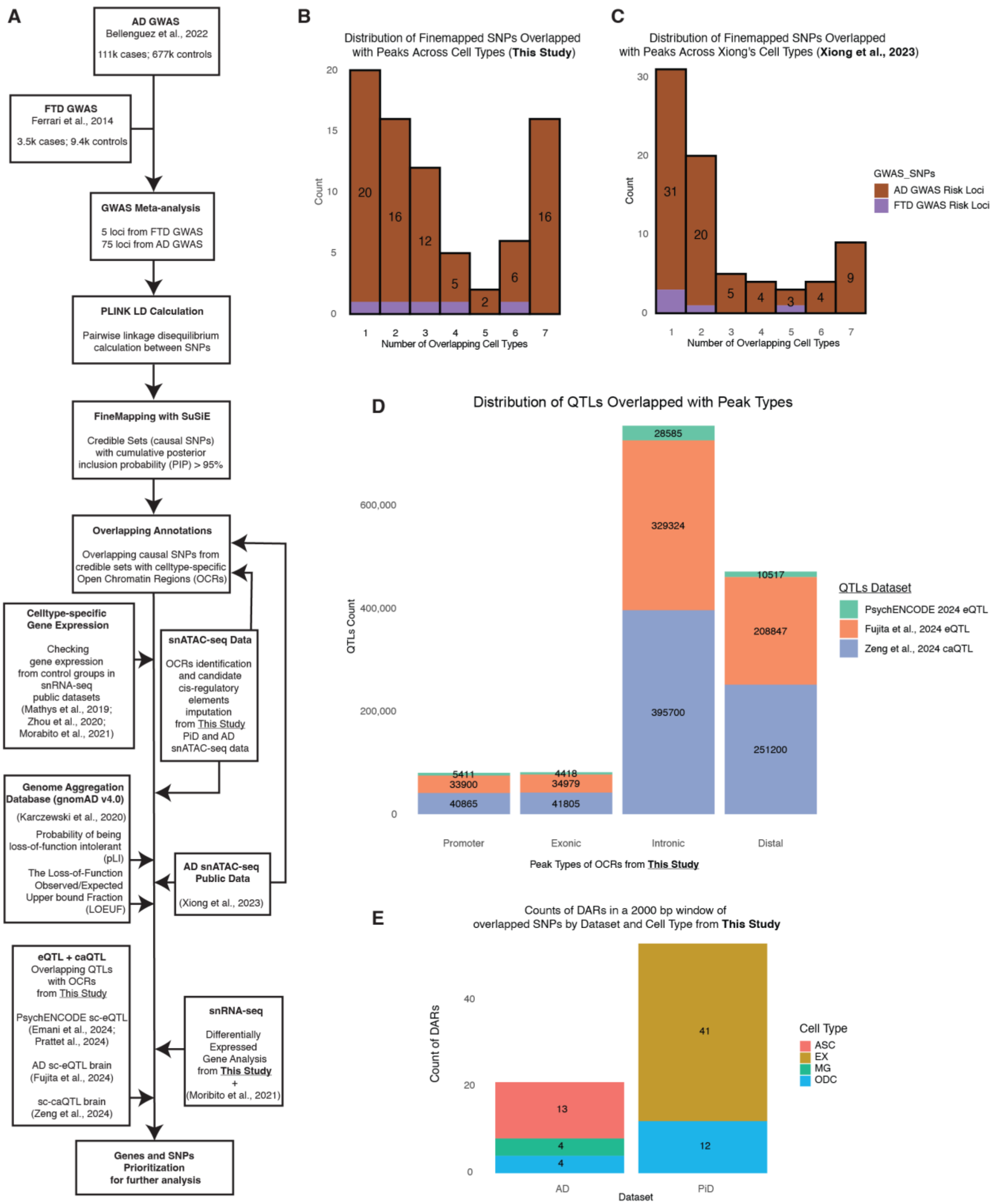

Fig. S3. Integration and validation of GWAS fine-mapped SNPs with open chromatin regions in FTD and AD.

(**A**) The schematic of analyses showing the summary of Frontotemporal Dementia (FTD) and Alzheimer's Disease GWAS meta-analyses, fine-mapping, and other data processing steps to

link causal SNPs to snATAC-seq accessible peaks in specific cell types. (**B**) Histogram of Overlapped Credible Sets with this study: Count vs. Overlapped Cell Types. It describes a histogram that displays the count of overlapped credible sets and their associated number of overlapped cell types. (**C**) Histogram of Overlapped Credible Sets with Xiong et al. (*27*): Count vs. Overlapped Cell Types. (**D**) Distribution of QTLs (*34*-*37*) overlapped with peak types from this study. (**E**) Counts of DARs in a 2000 bp window of overlapped SNPs by dataset and cell type from this study.

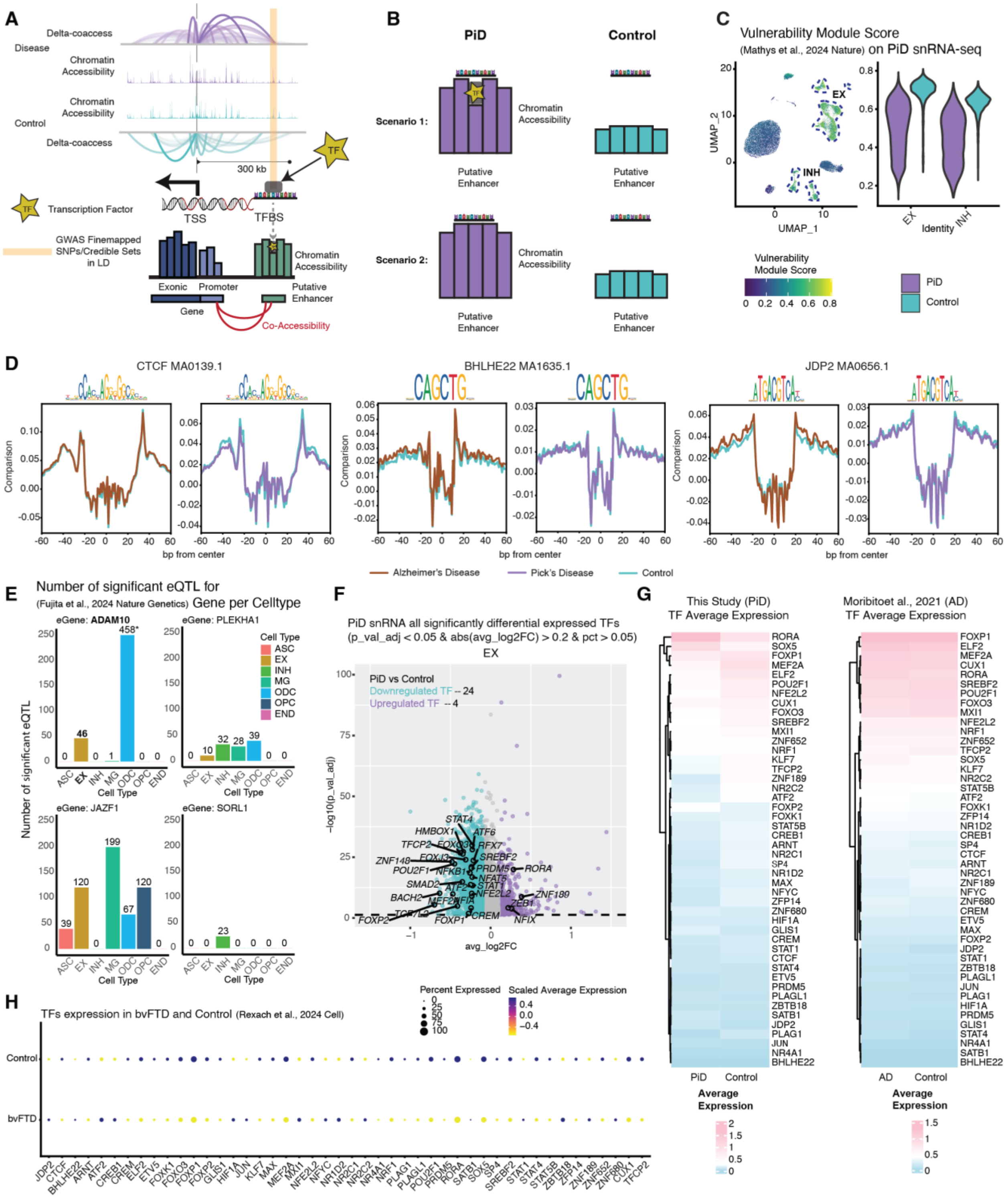

Fig. S4. EX transcription factor dynamics and differential expression in PiD and AD.

(A) Open chromatin co-accessibility plot with TF binding occupancy calculation. (B) Two open chromatin scenarios in PiD and control; scenario 1: Open chromatin with TF binding (footprint) in PiD; scenario 2: Open chromatin without TF binding (no footprint) in PiD. (**C**) Vulnerability module score (*60*) of neurons, EX and INH, in PiD. (**D**) Aggregated TF footprints of *CTCF*

(MA0139.1), *BHLHE22* (MA1635.1), and *JDP2* (MA0656.1) in AD and PiD. (**E**) Significant AD sc-eQTLs (p-value < 1×10ˆ-5) from Fujita et al. (*36*) by cell type. (**F**) PiD snRNA all significantly differential expressed TFs. (**G**) Heatmap of average expression difference of highlighted TFs and top selected differentially TFs regulated by highlighted TFs between PiD or AD with their matched control (FDR-adjusted p-value < 0.05). (**H**) Dotplot of differentially expressed TFs in Rexach et al. (*7*) bvFTD versus their respective controls.

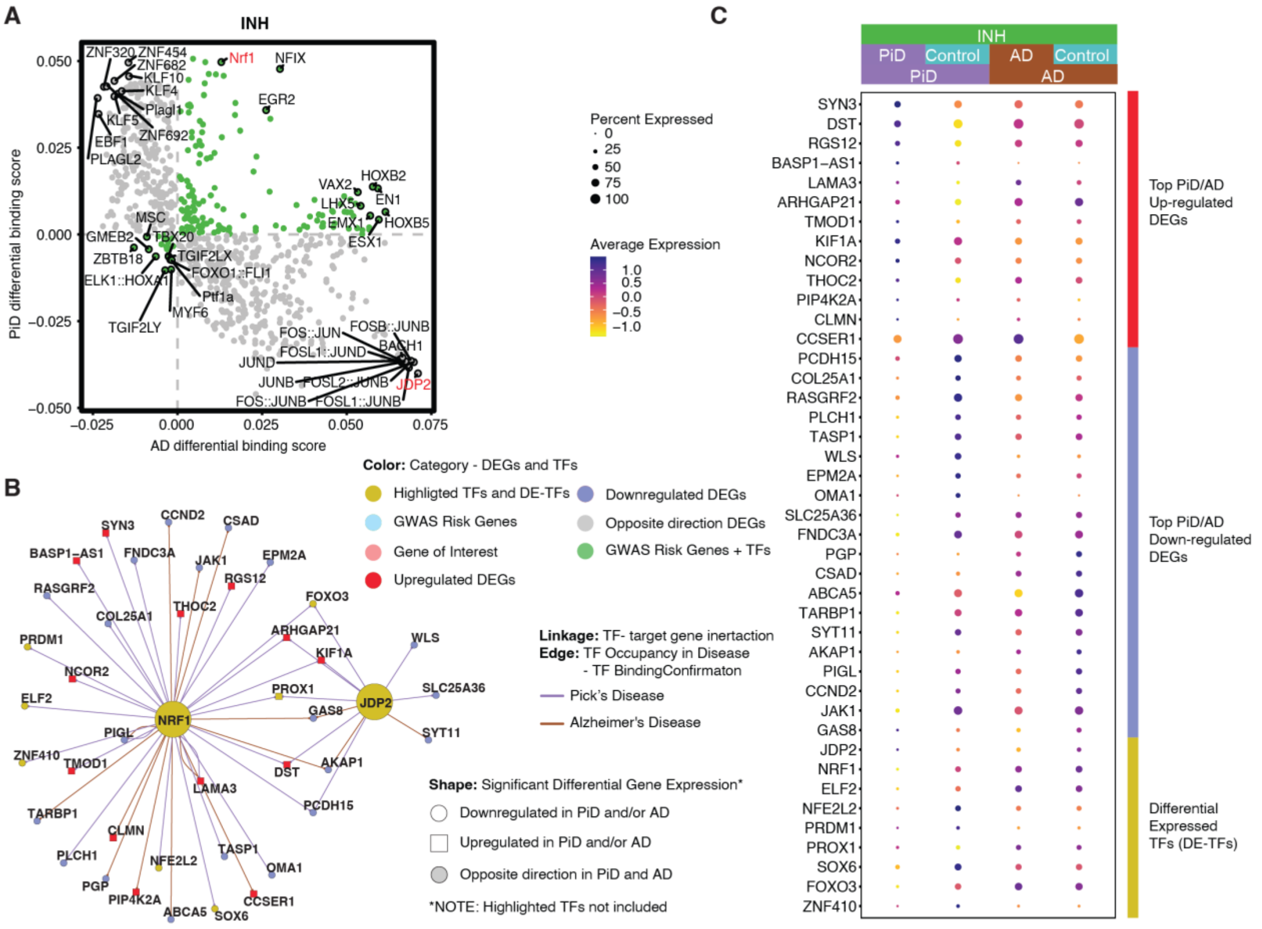

Fig. S5. INH TF binding difference and regulatory networks in PiD and AD.

(**A**) Genome-wide Tn5 bias-subtracted TF differential footprinting binding score of PiD and AD in INH. (**B**) *NRF1* and *JDP2* TF regulatory networks showing the predicted candidate target genes for INH. (**C**) Dot-plot of the differentially expressed genes and TFs in PiD and AD versus their respective controls.

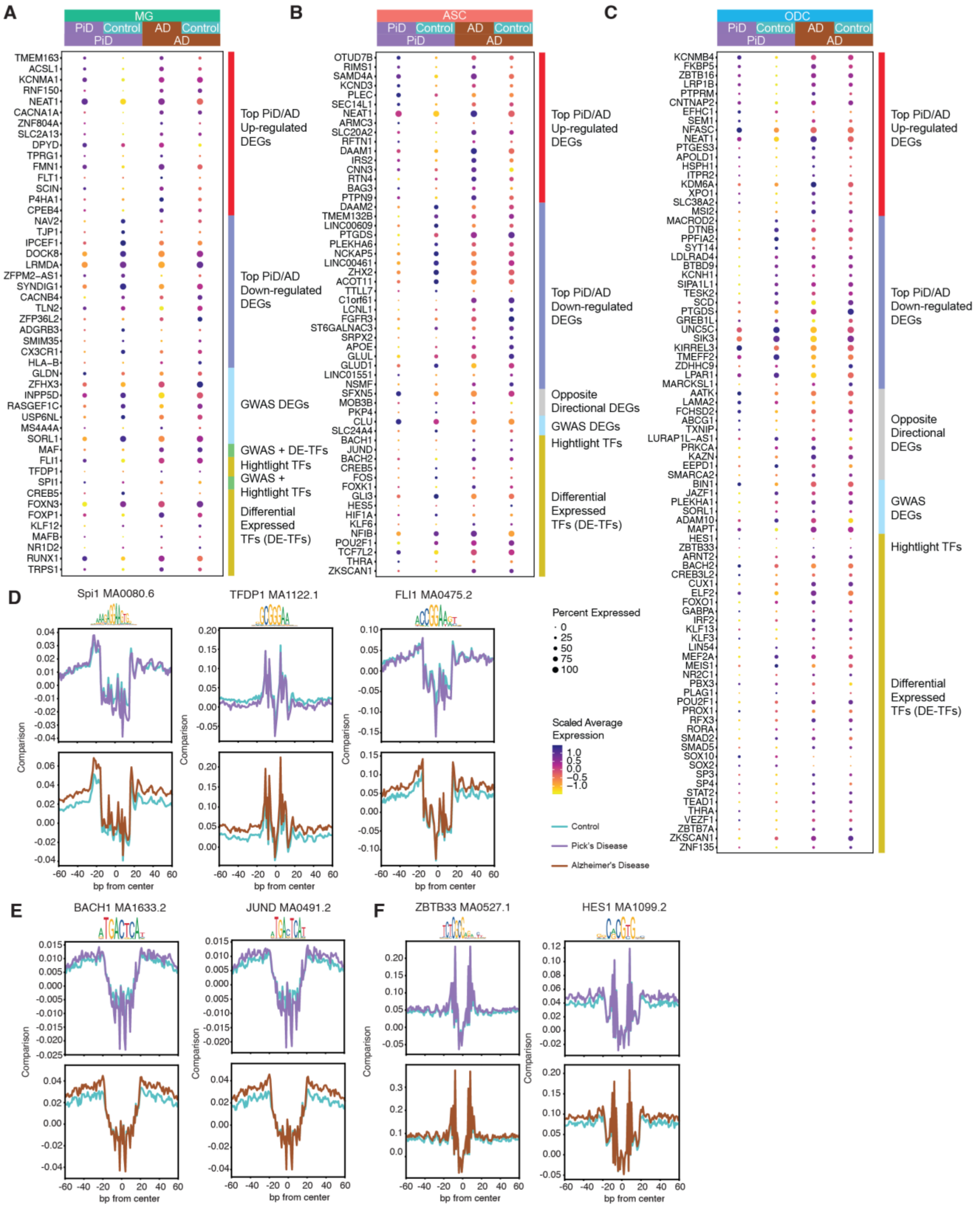

Fig. S6. Aggregated footprints of TFs in MG, ASC, and ODC.

(**A**, **B**, **C**) Dot-plot of the differentially expressed gene, differentially expressed GWAS risk genes, and TFs in PiD and AD versus their respective controls in MG (**A**), ASC (**B**), and ODC

(**C**). (**D**) Aggregated TF footprints of *Spi1* (MA0080.6), *TFDP1* (MA1122.1) and *FLI1* (MA0475.2) in PiD and AD. (**E**) Aggregated TF footprints of *BACH1* (MA1633.2) and *JUND* (MA0491.2) in PiD and AD. (**F**) Aggregated TF footprints of *ZBTB33* (MA0527.1) and *HES1* (MA1099.2) in PiD and AD.

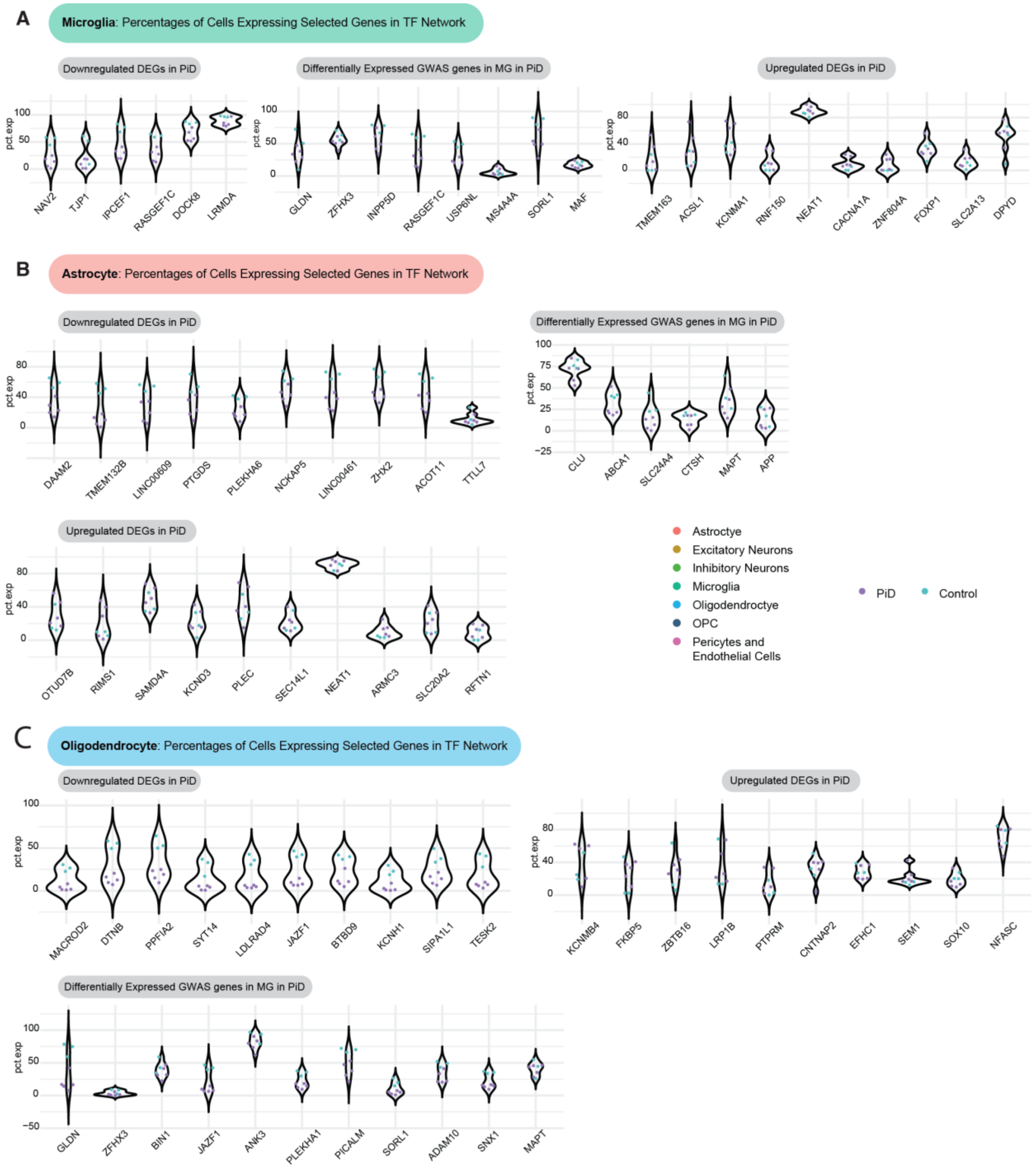

Fig. S7. Percentages of cells expressing selected genes in TF.

(**A**) Microglia: percentages of cells expressing selected genes in the TF network. (**B**) Astrocyte: percentages of cells expressing selected genes in the TF network. (**C**) Oligodendrocyte: percentages of cells expressing selected genes in the TF network.

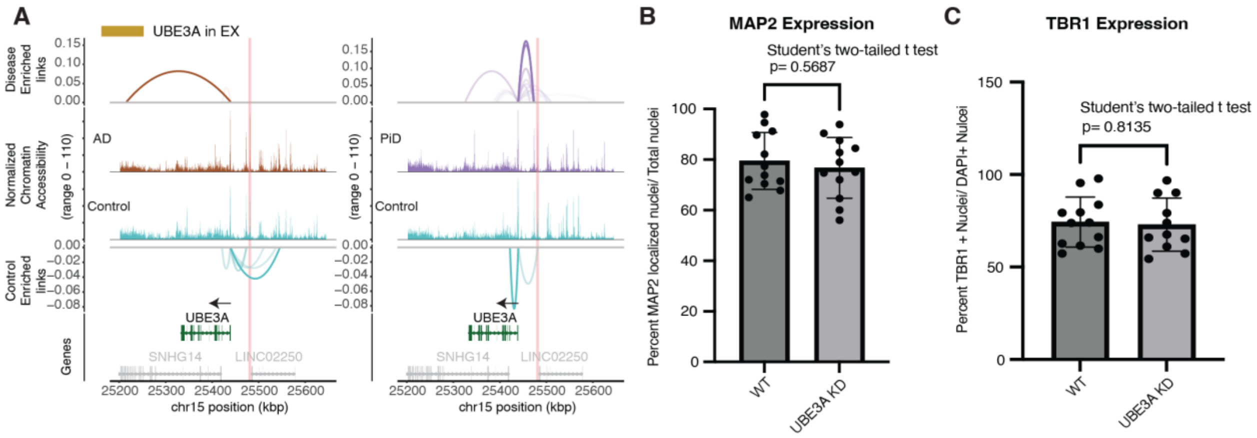

Fig. S8. UBE3A enhancer accessibility and iPSC-derived neurons confirmation.

(**A**) Delta co-accessibility of UBE3A and its open chromatin regions in EX for both AD and PiD with their corresponding controls. Highlighted regions in salmon represent CRISPR-edited enhancer regions to the UBE3A. (**B**) Quantification of DAPI nuclei colocalized with MAP2 expression, normalized to total nuclei. (**C**) Quantification of TBR1 positive nuclei normalized to total nuclei. Points represent individual images, n=4 per coverslip, n=1 coverslip per 3 differentiation replicates.

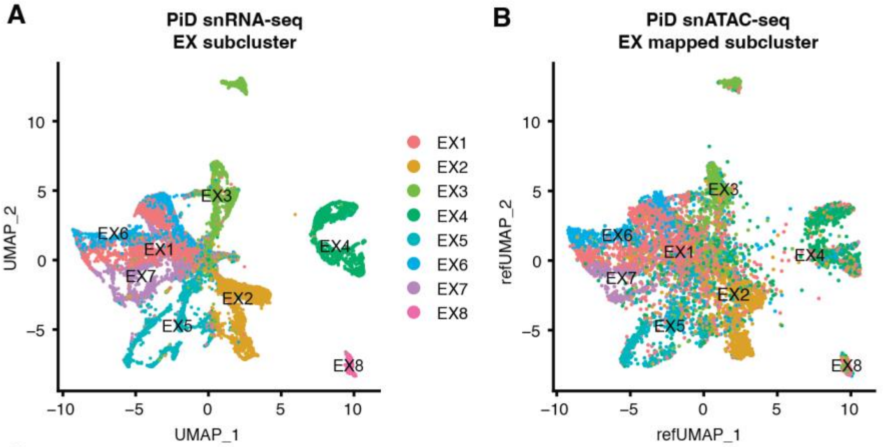

C

snRNA-seq			Comparison	Subclusters	snATAC-seq		
avg_log2FC	p_val	gene			UBE3A Enhancer	avg_log2FC	p_val
-0.2432003	1.5071E-09	UBE3A	PiDvsControl	EX1	chr15-25481385-25481885	1.36284651	0.00085508
-0.3906205	0.01084951	UBE3A	PiDvsControl	EX2	chr15-25481385-25481885	1.28636337	0.00368532
-0.2713886	0.04424086	UBE3A	PiDvsControl	EX3	chr15-25481385-25481885	1.03242802	0.05899418
-0.4626654	0.00884537	UBE3A	PiDvsControl	EX4	chr15-25481385-25481885	NA	NA
-0.3168027	0.00065383	UBE3A	PiDvsControl	EX5	chr15-25481385-25481885	1.62217714	0.00244501
-0.2595415	0.00059726	UBE3A	PiDvsControl	EX6	chr15-25481385-25481885	0.66093199	0.15996803
-0.1341428	0.02481595	UBE3A	PiDvsControl	EX7	chr15-25481385-25481885	1.67220543	0.00509698
-0.2773934	0.04398659	UBE3A	PiDvsControl	EX8	chr15-25481385-25481885	NA	NA

Fig. S9. Subclustering analysis on EX.

(**A**) UMAP of snRNA-seq EX subcluster. (**B**) UMAP of snATAC-seq EX subcluster. (**C**) Summary table of differential expression and accessibility analyses of EX subcluster.

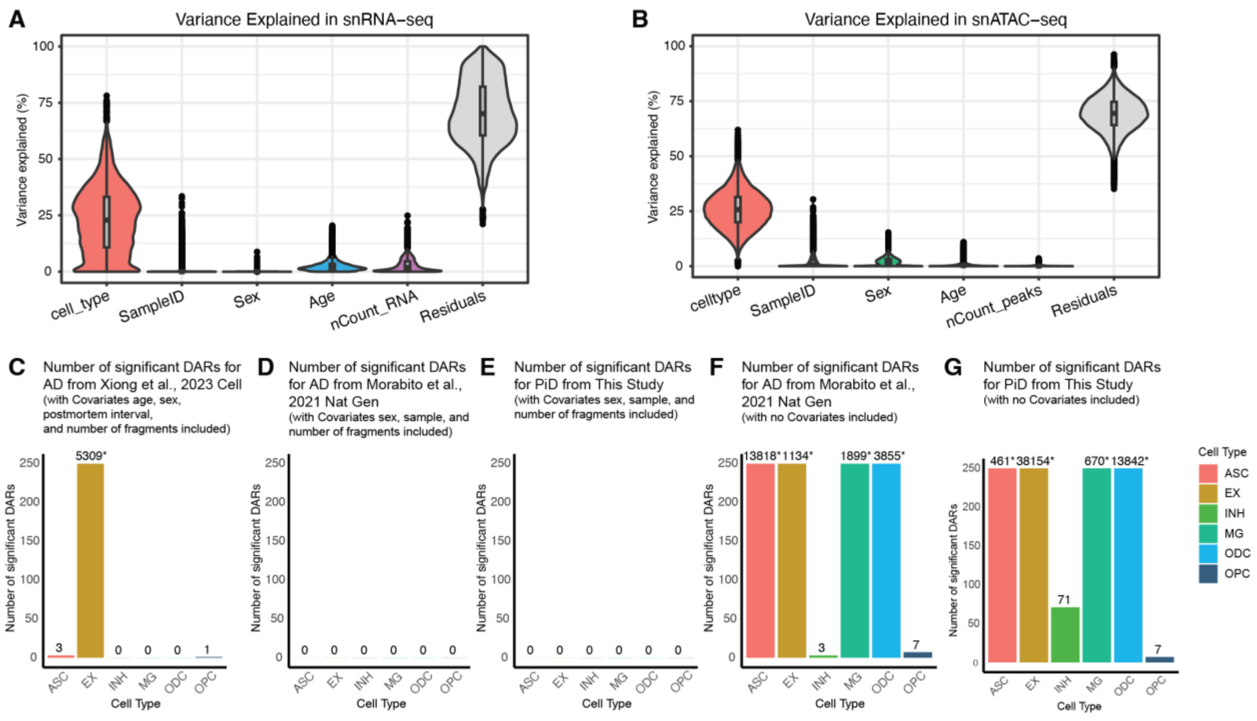

Fig. S10. Covariate partitioning and impact of covariate selection on the number of statistically significant differentially accessible regions (DARs). (**A**) Source of variation analysis showing the proportion of gene expression variance explained by each covariate used in the differential expression model in snRNA-seq. (**B**) Source of variation analysis showing the proportion of chromatin accessibility variance explained by each covariate used in the differential expression model in snATAC-seq. (**C**) Number of statistically significant DARs per cell type with covariates (age, sex, postmortem interval, number of fragments) for AD data from Xiong et al., 2023, Cell (27). DARs were reprocessed using the same covariates as in snRNA-seq. (**D**) Number of statistically significant DARs per cell type with covariates (sex, sample, number of fragments) for AD data from Morabito et al., 2021, Nature Genetics (17). (**E**) Number of statistically significant DARs per cell type with covariates (sex, sample, number of fragments) for PiD data from this study. No covariates were used in the original analysis. (**F**) Number of statistically significant DARs per cell type without any covariates for AD data from Morabito et al., 2021, Nature Genetics (17). (**G**) Number of statistically significant DARs per cell type without any covariates for PiD data from this study.

Table S1. Summary of supplementary tables providing metadata, marker analysis, and cell counts for PiD and AD datasets.
(**Table S1A**) Metadata for PiD and AD. (**Table S1B**) Results from snATAC-seq FindAllMarkers analysis on gene activity. (**Table S1C**) Cell counts for each cell type across snATAC-seq and snRNA-seq datasets. (**Table S1D**) Results from snRNA-seq FindAllMarkers analysis on gene expression.

Table S2. Summary of snATAC-seq peaks and differential accessibility regions (DARs) in PiD and AD datasets.
(**Table S2A**) Complete peak set of snATAC-seq for both PiD and AD. (**Table S2B**) Summary counts of peak type and biotype in PiD and AD DARs ($p < 0.05$). (**Table S2C**) DAR analysis for PiD vs Control (pct > 0.05), including all statistics, not just significant ones.

Table S3. Fine-mapping and GWAS enrichment analysis in PiD and AD datasets.
(**Table S3A**) Overlap of snATAC-seq peaks with SuSiE fine-mapped credible sets (PIP > 0.95) and Xiong et al., 2023 (*27*). (**Table S3B**) Complete list of SuSiE fine-mapped credible sets (PIP > 0.95). (**Table S3C**) GWAS gene enrichment analysis in PiD DGE.

Table S4. Differential gene expression (DGE) analysis in PiD and AD datasets.
(**Table S4A**) DGE results using MAST glm for PiD vs Control. (**Table S4B**) DGE results using MAST glm for AD vs Control.

Table S5. Human-gain enhancers (HGE) overlapped with snATAC-seq peaks in PiD and AD datasets.

Data S1. iPSC CRISPR KO Karyotype and Pluripotency Data Files.
(File 1) Project_Report_UBE3A__Swarup_6_30_22.pdf contains the design and results of the UBE3A CRISPR knockout experiment, including the knockout design strategy, experimental outcomes, quality control data for generated cell lines, and validation of iPSC characteristics. **(File 2)** Microarray REPORT CLG-46814_ADRC76.pdf is the microarray report for the parental iPSC line ADRC76, derived from fibroblasts and provided by the UCI Alzheimer's Disease Research Center (ADRC) iPSC Core. **(File 3)** Microarray REPORT CLG-46815_C40.pdf corresponds to Clone 40 derived from the ADRC76 iPSC line. **(File 4)** Microarray REPORT CLG-46816_C14.pdf corresponds to Clone 14 derived from the ADRC76 iPSC line.
