# Supplementary material for: Single-nucleus multi-omics identifies shared and distinct pathways in Pick’s and Alzheimer’s disease
Source: Sci Adv. 2025 Nov 12;11(46):eads7973. doi: 10.1126/sciadv.ads7973 (PMC12609060; doi:10.1126/sciadv.ads7973)
Supplement: Supplementary file 3 — Data S1 [file sciadv.ads7973_data_s1.zip › ads7973_data_s1.zip!/DataS1_iPSC_crispr_ko_karyotype_pluripotency/Microarray REPORT CLG-46816_C14.pdf]

www.clgenetics.com
510 Charmany Drive • #254–255 • Madison, Wisconsin 53719 • 608-441-8160

CLG Microarray Test Results

Cell Line ID: ADRC76 C-14 p17 **Lab #:** CLG-46816 **Date received:** 6/09/22 **Date Reported:** 6/29/22

Contact Person: Anita Lakatos **PI:** Mathew Blurton-Jones **Institute:** University of California Irvine

Test Code: aCGH 100 **Email:** scrs-crispr@uci.edu **PO #:** 309156

Mailing Address: 845 Health Sciences Road, 1200 Gross Hall, Irvine, CA 92617

Sample Type: Human iPSC Frozen Cells **dsDNA Concentration:** 394 ng/µl **Total dsDNA:** 5.1 µg

Sex: Male **260/280 (1.7-1.9):** 1.87 **260/230 (≥1.0):** 2.39 **Array Type:** Agilent 60K Standard aCGH

Array ID Number: 252192456334_2_3 **Human Assembly:** hg38 **Reference DNA:** Agilent Euro Male

---

Quality Control

A sufficient amount of high quality genomic DNA, as determined by UV spec. (Nanodrop), fluorometer (Qubit) and Agarose Gel analysis, was extracted from cell line ADRC76 C-14 p17 and passed our internal quality standards for aCGH labeling.

aCGH Probes (PASS/FAIL): Pass

Experimental Deviations: None

Results:

See attached sheets for Tabular and Graphical presentation of microarray results.

Variants are considered provisional until confirmed by another technique. For further confirmation of a particular variant, CLG recommends using Karyotyping (variants >5Mb) or FISH (variants>200Kb).

Sample Information

Red Sample	:	CLG-46816
Array ID	:	252192456334_2_3
SignedOff by	:	CLG\jpanella

Amp/Gain/Loss/Del Intervals Table

Chr	Start-Stop(bp)	Cytoband	Size(kb)	#Probes	Amp/Gain/ Loss/Del	Annotations
chr10	66405787-66684136	q21.3	278.35	7	-0.953	CTNNA3
chr21	42539595-42816565	q22.3	276.971	8	0.514	SLC37A1, LINC01671, PDE9A, LINC01668

Amp=Amplification Del=Deletion

Total Amp/Gain/Loss/Del Intervals: 2

Chromosome Views (Amp/Del)

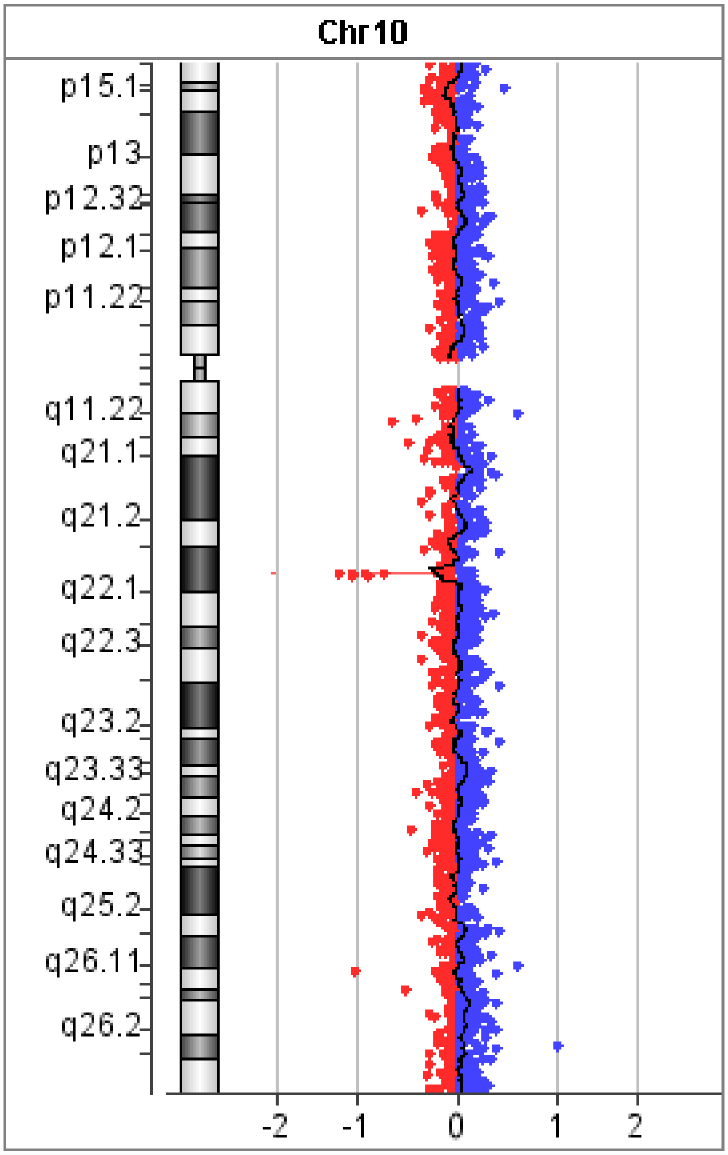

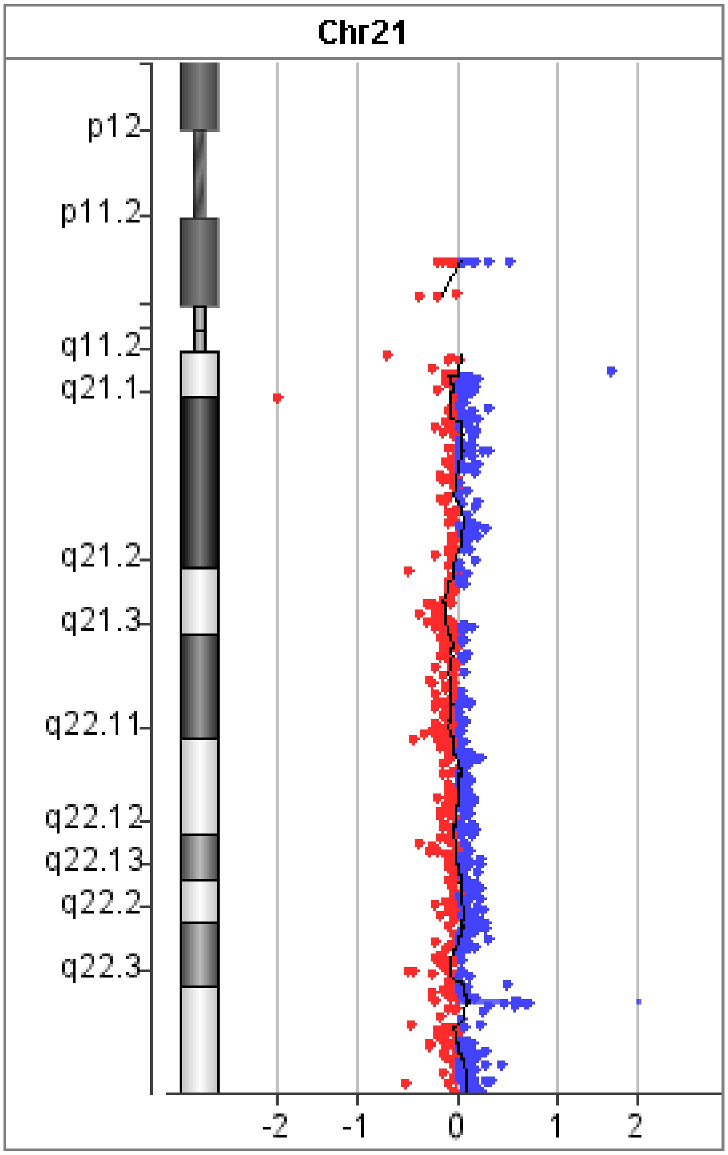
Chr21
p12
p11.2
q11.2
q21.1
q21.2
q21.3
q22.11
q22.12
q22.13
q22.2
q22.3
-2
-1
0
1
2
