# Supplementary material for: Single-nucleus multi-omics identifies shared and distinct pathways in Pick’s and Alzheimer’s disease
Source: Sci Adv. 2025 Nov 12;11(46):eads7973. doi: 10.1126/sciadv.ads7973 (PMC12609060; doi:10.1126/sciadv.ads7973)
Supplement: Supplementary file 3 — Data S1 [file sciadv.ads7973_data_s1.zip › ads7973_data_s1.zip!/DataS1_iPSC_crispr_ko_karyotype_pluripotency/Project_Report_UBE3A__Swarup_6_30_22_age info masked.pdf]

CRISPR Gene Editing Core
Stem Cell Research Center
University of California, Irvine
1200 (Bay 3, North) Gross Hall
845 Health Sciences Road
Irvine, CA 92697-1705
Email: srcr-crispr@uci.edu

6/30/2022

Dear Dr. Swarup,

As requested, we have successfully removed approximately 3 kb regulatory region of the UBE3A gene on Chr 15. Additionally, we have concluded our quality control assessment and are pleased to provide you with an edited homozygous cell line (C-14). Please note that we obtained two modified cell lines, and the second cell line (C-40) is also available to you for an additional small fee if you decide to purchase.

##
############# ***Project Report*** #############
##
Project Title: Deletion of ~3 kb UBE3A [*Homo sapiens*] enhancer region on Chr 15
PI: Vivek Swarup Ph.D.
Department: Neurobiology and Behavior
School: Biological Sciences
University California, Irvine
Contact: vswarup@uci.edu

Date of Project Completion: 6/30/2022

Project Summary
Deletion of approx. 3 kb regulatory region (highlighted in blue) of the UBE3A gene based on the coordinates provided by Dr. Swarup.

Coordinates of Dr. Swarup: chr15:25,479,200-25,482,595

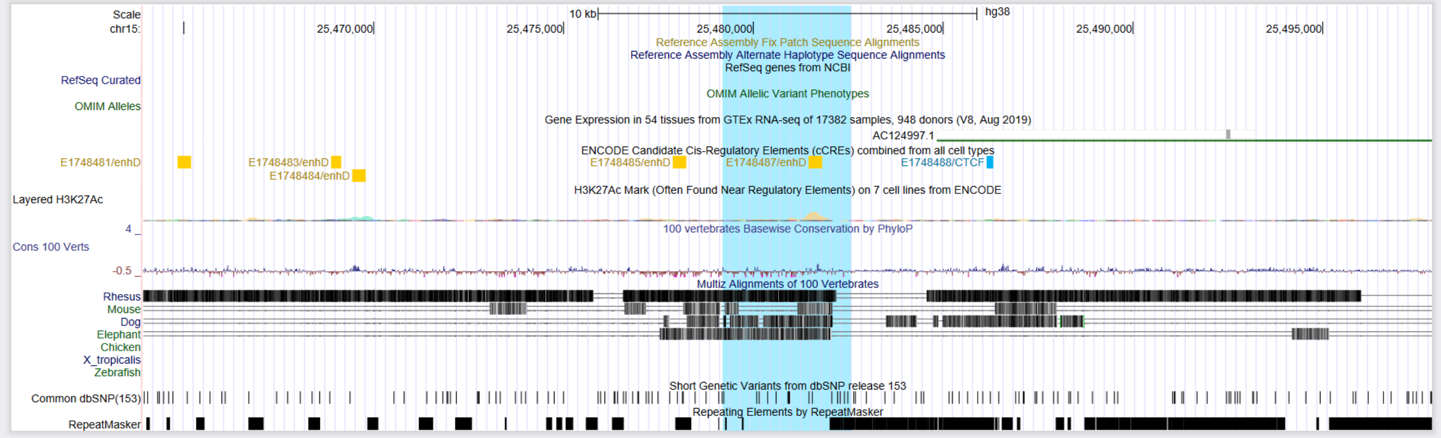

Design Strategy

1.Cell Line

ADRC76 iPS cell line was choosen for genome editing (origin: fibroblast; passage number:14).

2. CRISPR guide RNA

Two guide RNAs flanking the regulatory region were designed and delivered as an RNP (guides + Cas9) complex to the cells via electroporation.

cRNA_1 (upstream)	5'-TGTTTTTTAGACACCTGCGC-3'
cRNA_2 (downstream)	5'-AAACATTTTACAGACTCCAG-3'

Results

1. **Genomic Alteration**

The target region was subjected to PCR amplification, and the editing and clonality were verified through several rounds of visual inspection and Sanger sequencing.

Forward PCR Primer: UBE3A_F: 5'-TACAGAGAGAACTACATCCTCCACT-3'
Reverse PCR Primer: UBE3A_R: 5'AAAACATGAGCACACACTCATCTAC-3'

Product Size Wild type: ~ 3200 bps
Product size Edited: ~ 400 bp

M Wild type cells/Edited cells

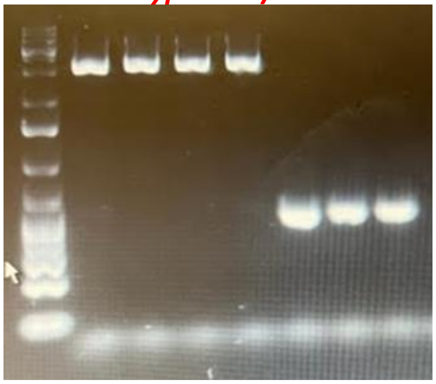

Edited cell lines (C-14 and C-40) at various times during the process

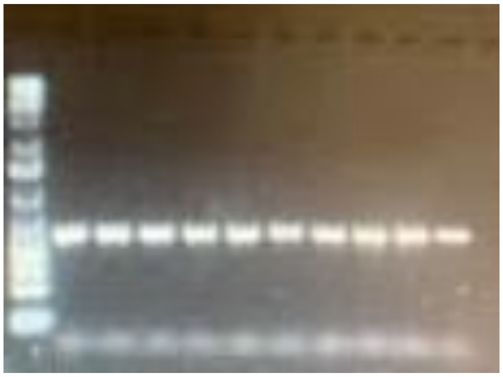

Sanger Sequencing (Retrogen Inc.)

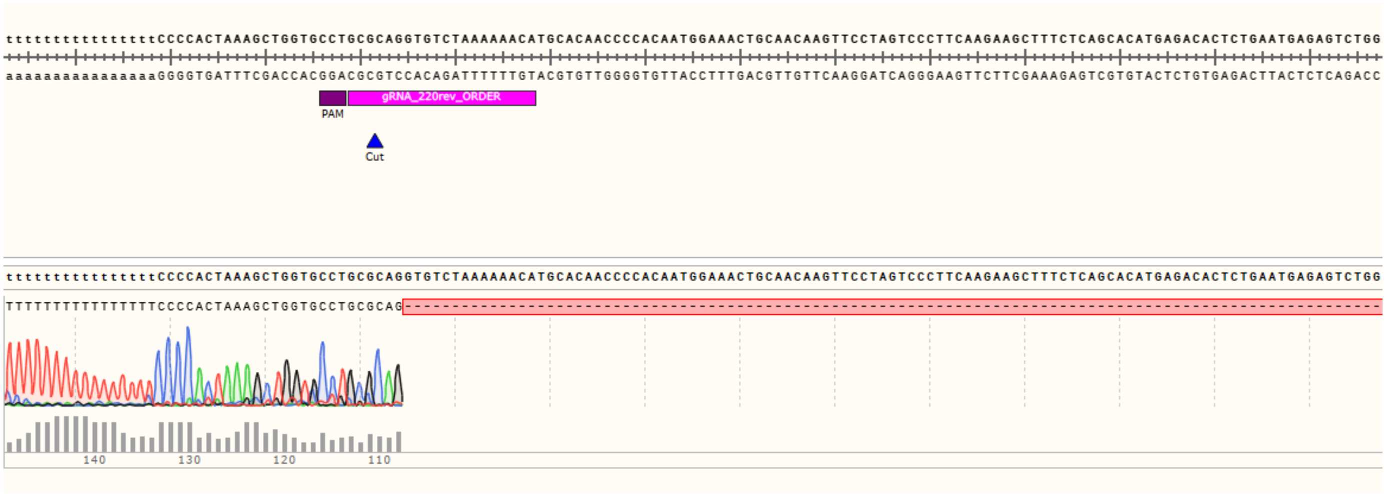

Continue….

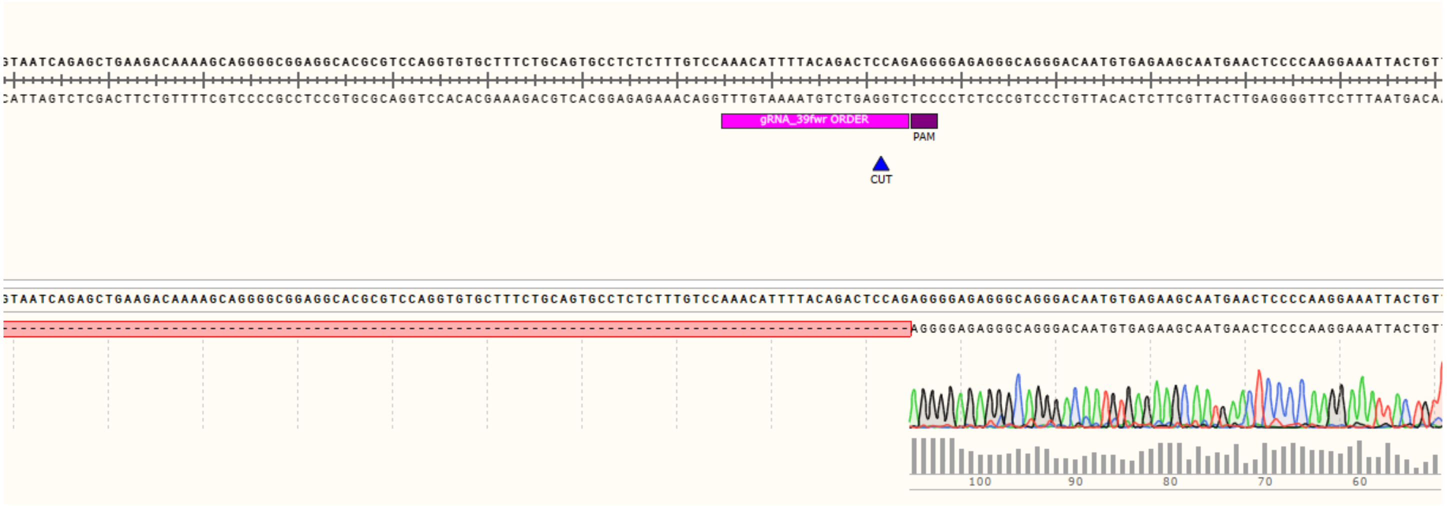

Sanger sequencing suggests that the cell line is heterozygous for the deletion with approximately one bp difference at the upstream cut - the deletion is not identical for both chromosomes (~1 pb difference), which were expected given the size of the deletion relying on NHEJ-Based DNA Repair.

In addition, the edited sequence was aligned to the human genome (hg38) in UCSC Genome Browser using BLAT function implemented in the genome browser. The edit region is indicated as “YourSeq: Your sequence

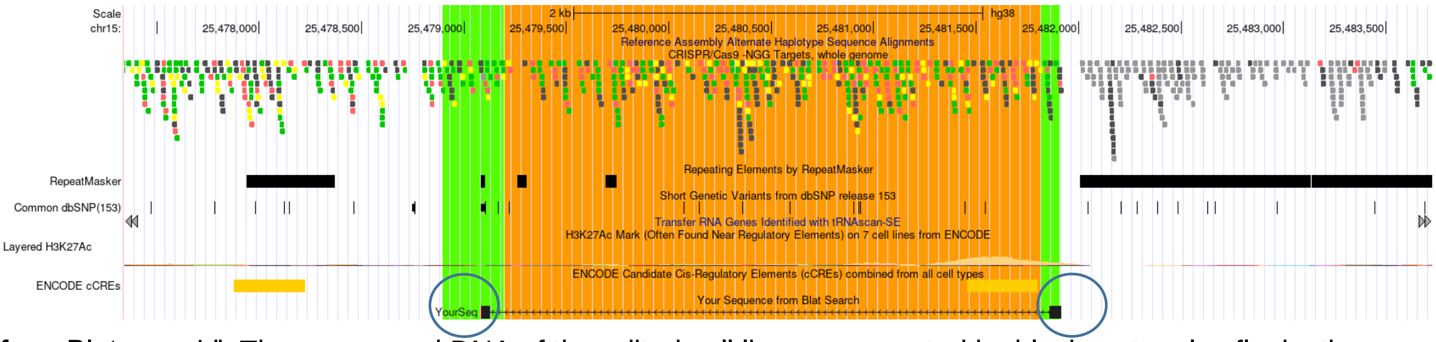

from Blat search”. The sequenced DNA of the edited cell lines represented by black rectangles flanks the deleted region (coordinates: chr15:25,479,128-25,481,855)

T7 Endonuclease I -based Mutation Detection Assay (Qualitative Assessment)

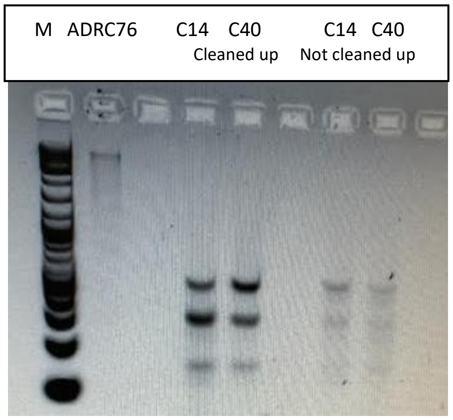

T7 qualitative assignment substantiates the result of the Sanger sequencing: both chrs are edited as desired; however, the deletions on both chromosomes differ approximately ~1 pb upstream

2. *CellLine Quality Check*

A. *Contamination*

Contamination has been tested several times during the process.

The latest results before sending to Cell Line Genetics.

I. Mycoplasma test (MycoAlert PLUS Mycoplasma Detection Kit from Lonza) of the edited cell lines yielded negative outcome.

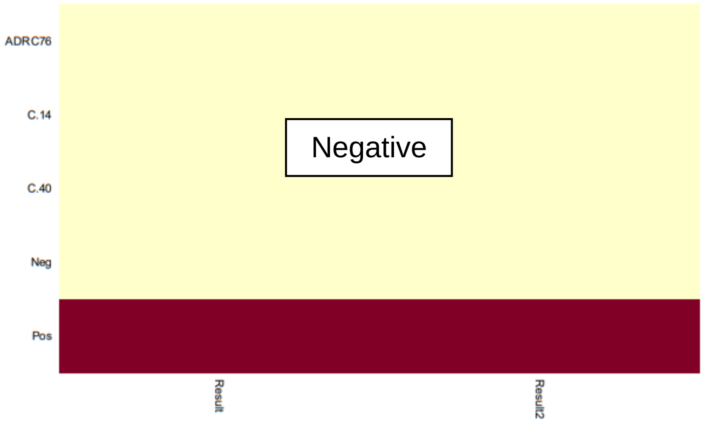

II. Onar Bacteria Detection test (The kit can detect more than 45 bacteria genera)

Test results: Negative

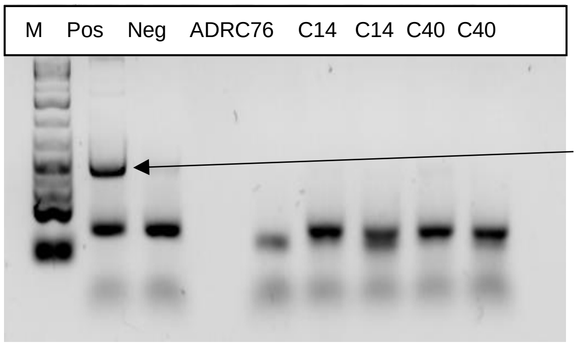

A band in correspondence of the target (ca. 460 bp) implies the presence of a bacterial contamination.

B. iPSC morhology

The edited cell lines exibit iPSC morphology (cobblestone, tightly packed cells with a high ratio of nucles/cytolasms, sharpe colony edges with no sign of differentiation)

ADRC76

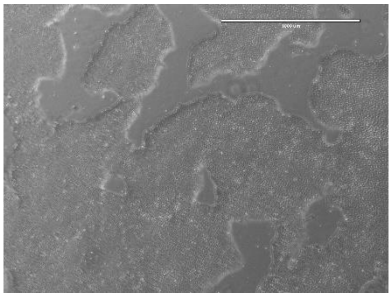

C-14

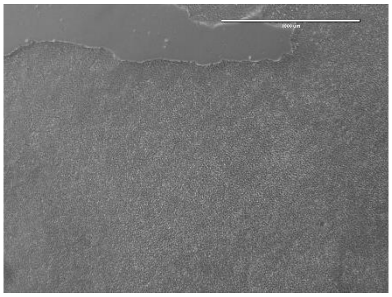

C-40

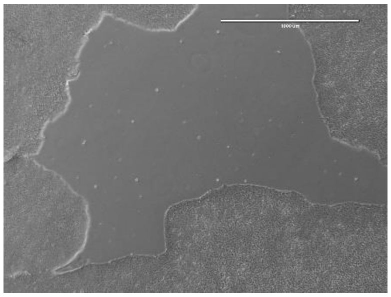

C. Cell Line Genetics (CLG) Karyotype Report

Karyotype normal with no additional alterations as assessed by array CGH to the parental line (the report is attached separtely)

##

Please let us know if you have any questions regarding our report and the date when you wish to transfer the edited cell line!

Warm regards,

Anita Lakatos
CRISPR Gene Editing Core
scrc-crispr@uci.edu
