# Supplementary material for: Single-nucleus multi-omics identifies shared and distinct pathways in Pick’s and Alzheimer’s disease
Source: Sci Adv. 2025 Nov 12;11(46):eads7973. doi: 10.1126/sciadv.ads7973 (PMC12609060; doi:10.1126/sciadv.ads7973)
Supplement: Supplementary file 3 — Data S1 [file sciadv.ads7973_data_s1.zip › ads7973_data_s1.zip!/DataS1_iPSC_crispr_ko_karyotype_pluripotency/Microarray REPORT CLG-46814_ADRC76.pdf]

CLG Microarray Test Results

Cell Line ID: ADRC76 p17 **Lab #:** CLG-46814 **Date received:** 6/09/22 **Date Reported:** 6/29/22

Contact Person: Anita Lakatos **PI:** Mathew Blurton-Jones **Institute:** University of California Irvine

Test Code: aCGH 100 **Email:** scrs-crispr@uci.edu **PO #:** 309156

Mailing Address: 845 Health Sciences Road, 1200 Gross Hall, Irvine, CA 92617

Sample Type: Human iPSC Frozen Cells **dsDNA Concentration:** 366 ng/µl **Total dsDNA:** 4.6 µg

Sex: Male **260/280 (1.7-1.9):** 1.85 **260/230 (≥1.0):** 2.14 **Array Type:** Agilent 60K Standard aCGH

Array ID Number: 252192456334_2_1 **Human Assembly:** hg38 **Reference DNA:** Agilent Euro Male

---

Quality Control

A sufficient amount of high quality genomic DNA, as determined by UV spec. (Nanodrop), fluorometer (Qubit) and Agarose Gel analysis, was extracted from cell line ADRC76 p17 and passed our internal quality standards for aCGH labeling.

aCGH Probes (PASS/FAIL): Pass

Experimental Deviations: None

Results:

See attached sheets for Tabular and Graphical presentation of microarray results.

Variants are considered provisional until confirmed by another technique. For further confirmation of a particular variant, CLG recommends using Karyotyping (variants >5Mb) or FISH (variants>200Kb).

Sample Information

Red Sample	:	CLG-46814
Array ID	:	252192456334_2_1
SignedOff by	:	CLG\jpanella

Amp/Gain/Loss/Del Intervals Table

Chr	Start-Stop(bp)	Cytoband	Size(kb)	#Probes	Amp/Gain/ Loss/Del	Annotations
chr7	121634411-122994947	q31.32	1,360.537	35	0.275	PTPRZ1, AASS, FEZF1, FEZF1-AS1, CADPS2, RNF133, RNF148, TAS2R16
chr10	66405787-66684136	q21.3	278.35	7	-0.959	CTNNA3
chr21	42539595-42771891	q22.3	232.297	7	0.577	SLC37A1, LINC01671, PDE9A

Amp=Amplification Del=Deletion

Total Amp/Gain/Loss/Del Intervals: 3

Chromosome Views (Amp/Del)

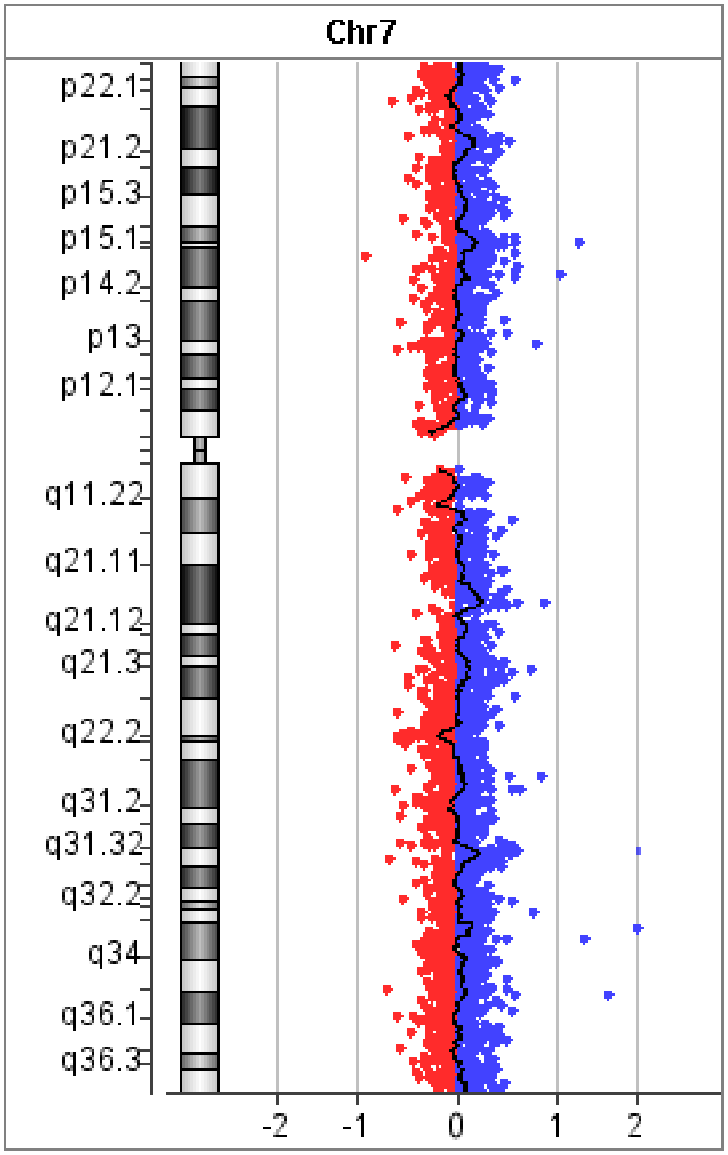

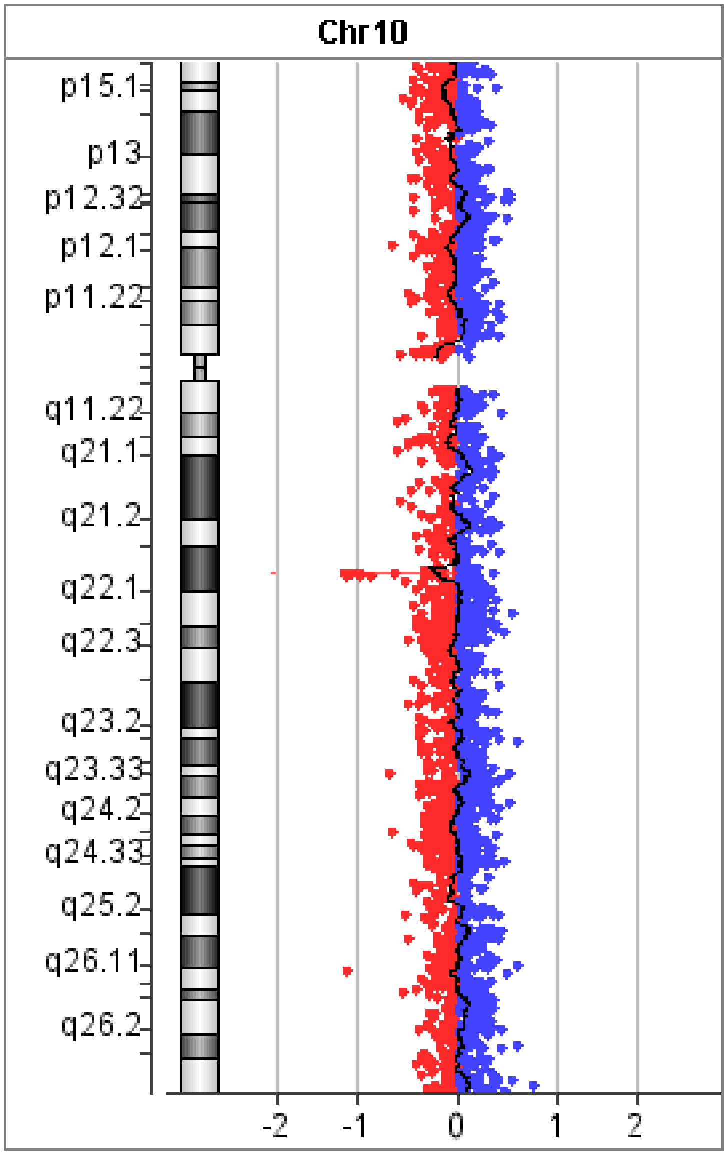

Chr10
p15.1
p13
p12.32
p12.1
p11.22
q11.22
q21.1
q21.2
q22.1
q22.3
q23.2
q23.33
q24.2
q24.33
q25.2
q26.11
q26.2
-2
-1
0
1
2

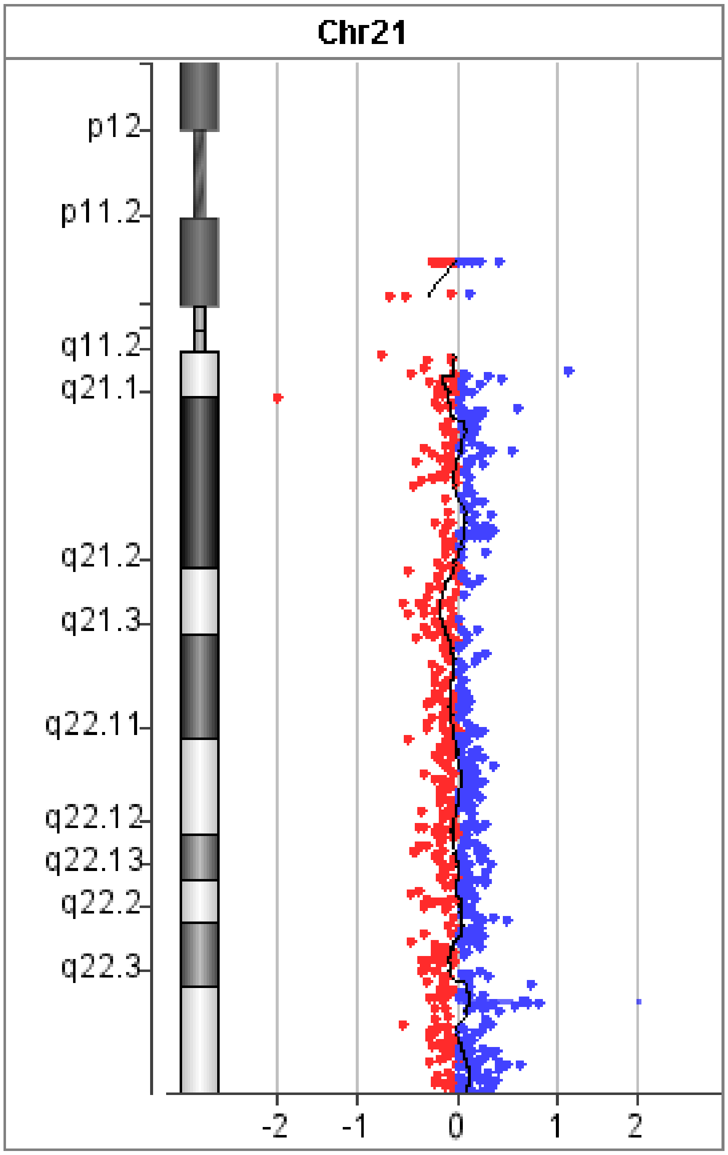
Chr21
p12
p11.2
q11.2
q21.1
q21.2
q21.3
q22.11
q22.12
q22.13
q22.2
q22.3
-2
-1
0
1
2
