# Supplementary material for: Single-nucleus multi-omics identifies shared and distinct pathways in Pick’s and Alzheimer’s disease
Source: Sci Adv. 2025 Nov 12;11(46):eads7973. doi: 10.1126/sciadv.ads7973 (PMC12609060; doi:10.1126/sciadv.ads7973)
Supplement: Supplementary file 3 — Data S1 [file sciadv.ads7973_data_s1.zip › ads7973_data_s1.zip!/DataS1_iPSC_crispr_ko_karyotype_pluripotency/Microarray REPORT CLG-46815_C40.pdf]

Cell Line GENETICS
www.clgenetics.com
510 Charmany Drive • #254–255 • Madison, Wisconsin 53719 • 608-441-8160

CLG Microarray Test Results

Cell Line ID: ADRC76 C-40 p17 **Lab #:** CLG-46815 **Date received:** 6/09/22 **Date Reported:** 6/29/22

Contact Person: Anita Lakatos **PI:** Mathew Blurton-Jones **Institute:** University of California Irvine

Test Code: aCGH 100 **Email:** scrs-crispr@uci.edu **PO #:** 309156

Mailing Address: 845 Health Sciences Road, 1200 Gross Hall, Irvine, CA 92617

Sample Type: Human iPSC Frozen Cells **dsDNA Concentration:** 350 ng/µl **Total dsDNA:** 4.4 µg

Sex: Male **260/280 (1.7-1.9):** 1.86 **260/230 (≥1.0):** 2.26 **Array Type:** Agilent 60K Standard aCGH

Array ID Number: 252192456334_2_2 **Human Assembly:** hg38 **Reference DNA:** Agilent Euro Male

---

Quality Control

A sufficient amount of high quality genomic DNA, as determined by UV spec. (Nanodrop), fluorometer (Qubit) and Agarose Gel analysis, was extracted from cell line ADRC76 C-40 p17 and passed our internal quality standards for aCGH labeling.

aCGH Probes (PASS/FAIL): Pass

Experimental Deviations: None

Results:

See attached sheets for Tabular and Graphical presentation of microarray results.

Variants are considered provisional until confirmed by another technique. For further confirmation of a particular variant, CLG recommends using Karyotyping (variants >5Mb) or FISH (variants>200Kb).

Sample Information

Red Sample	:	CLG-46815
Array ID	:	252192456334_2_2
SignedOff by	:	CLG\jpanella

Amp/Gain/Loss/Del Intervals Table

Chr	Start-Stop(bp)	Cytoband	Size(kb)	#Probes	Amp/Gain/Loss/Del	Annotations
chr7	121634411-123125667	q31.32	1,491.257	38	0.276	PTPRZ1, AASS, FEZF1, FEZF1-AS1, CADPS2, RNF133, RNF148, TAS2R16, SLC13A1
chr10	36991435-38139119	p11.21 - p11.1	1,147.685	20	-0.326	ANKRD30A, LINC00993, MTRNR2L7, ZNF248, ZNF33BP1, ZNF25, ZNF33A, ZNF37A
chr10	66405787-66684136	q21.3	278.35	7	-0.959	CTNNA3
chr21	42539595-42771891	q22.3	232.297	7	0.550	SLC37A1, LINC01671, PDE9A

Amp=Amplification Del=Deletion

Total Amp/Gain/Loss/Del Intervals: 4

Chromosome Views (Amp/Del)

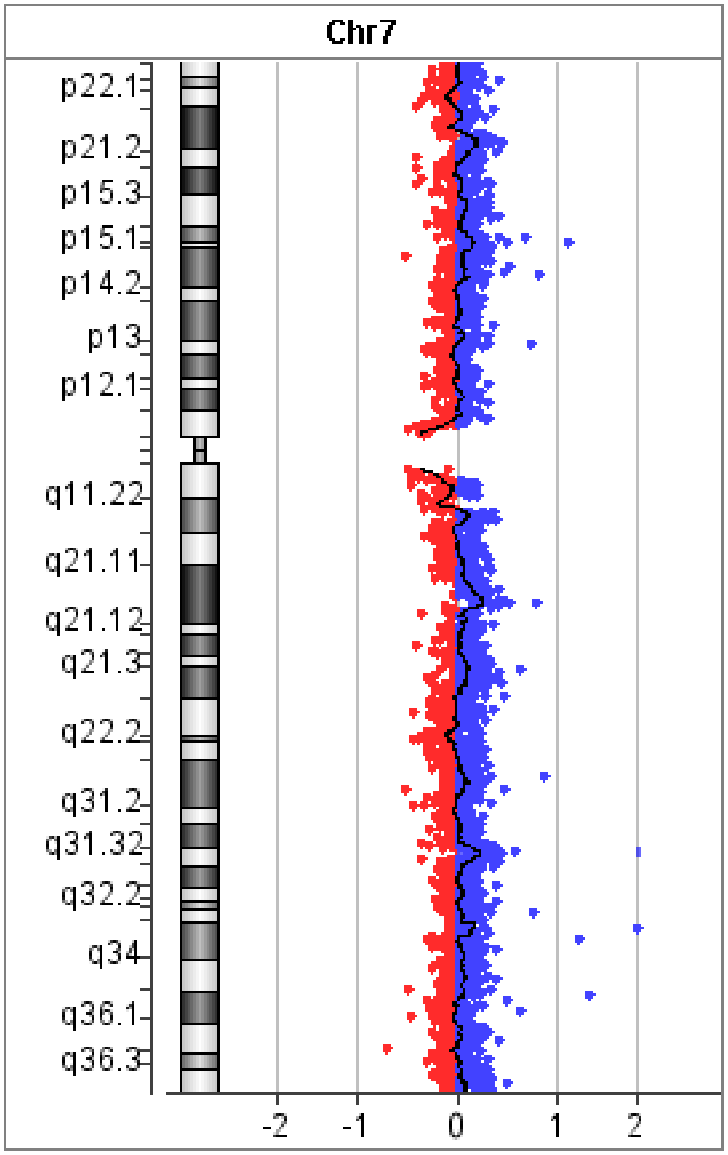

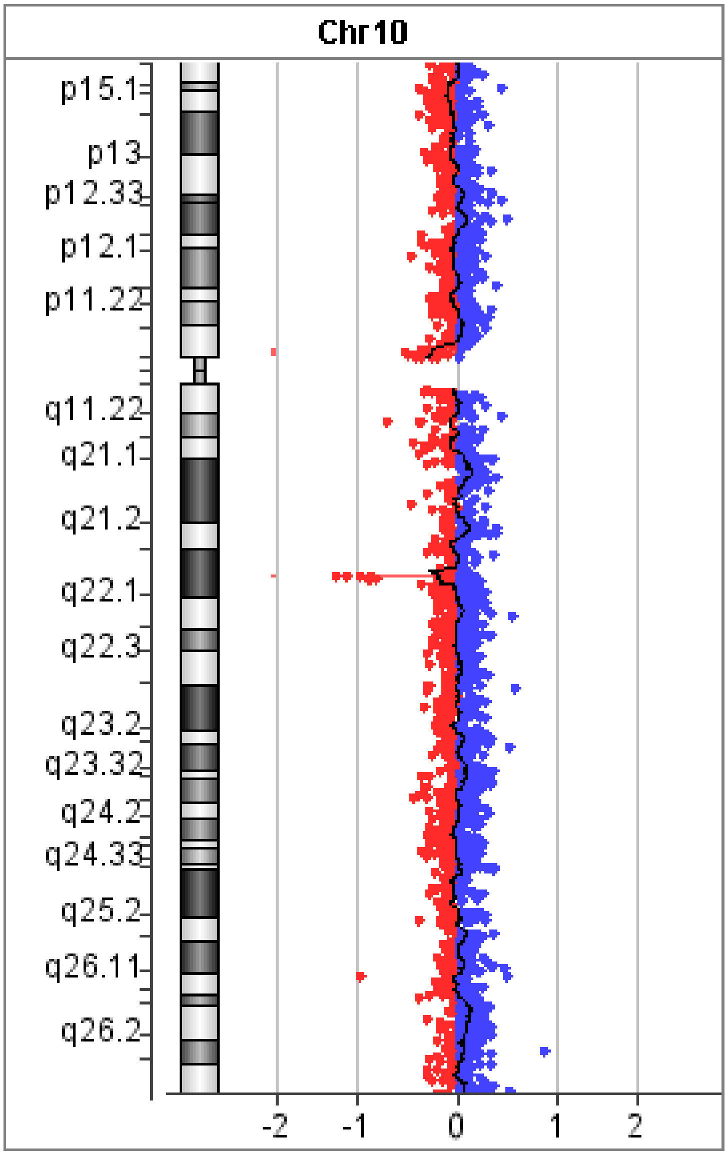
Chr10
p15.1
p13
p12.33
p12.1
p11.22
q11.22
q21.1
q21.2
q22.1
q22.3
q23.2
q23.32
q24.2
q24.33
q25.2
q26.11
q26.2
-2
-1
0
1
2

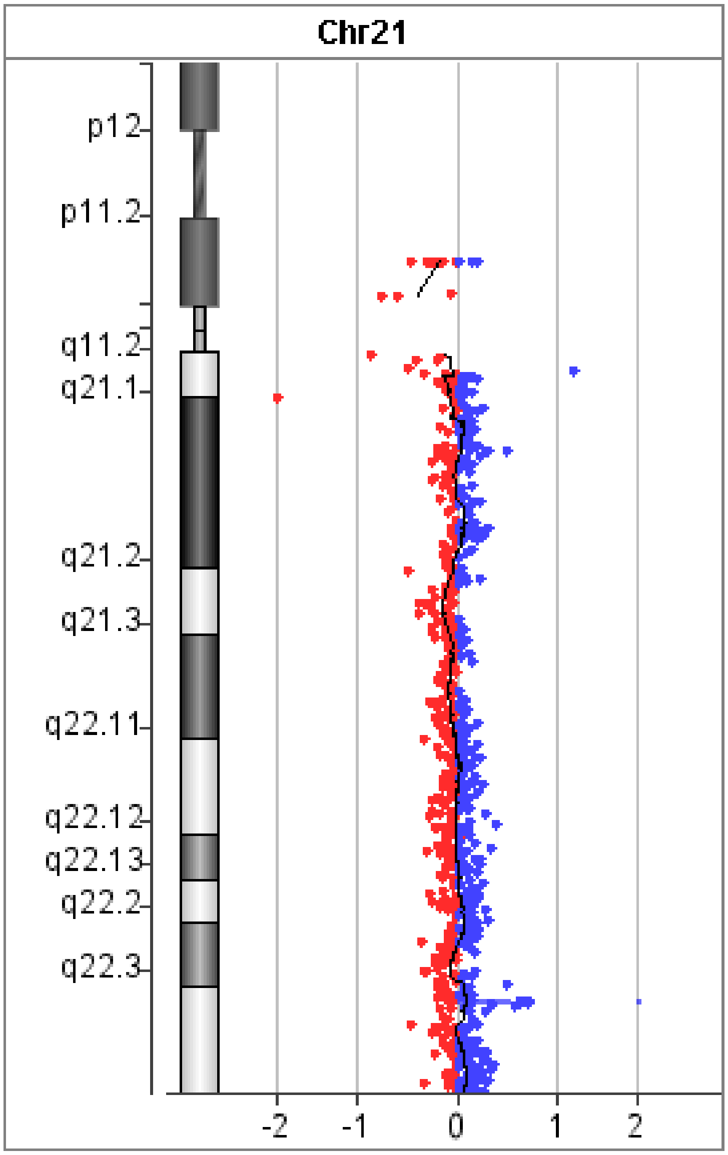
Chr21
p12
p11.2
q11.2
q21.1
q21.2
q21.3
q22.11
q22.12
q22.13
q22.2
q22.3
-2
-1
0
1
2
